# Supplementary material for: Promoting equity on licensing exams: Test accommodations for medical students with diabetes
Source: PLoS One. 2026 Feb 20;21(2):e0340975. doi: 10.1371/journal.pone.0340975 (PMC12923048; doi:10.1371/journal.pone.0340975)
Supplement: S1 File — (PDF) [file pone.0340975.s001.pdf]

REVIEW OF SAFEGUARDS FOR HUMAN SUBJECTS

American Institutes for Research
1000 Thomas Jefferson Street, NW
Washington, DC 20007

Institutional Review Board
IRB00000436

Project number: EX00398
Project Director/Proposal Author: Mark Raymond
Project/Proposal title: **NBME Institutional Research Plan**

1. Type of review:

(Check one)
☒ Expedited review
☐ Full IRB review

(Check one)
☒ Initial review
☐ Scheduled re-review (e.g., annual)
☐ Requested re-review (e.g., new data collection component, research plan change)

2. Review determination:

After reviewing the above *project* the Institutional Review Board (or member signing below) has determined the following:

☒ **Determination of Exemption: the project is exempt from further IRB review because it does not constitute research or because it does not involve human subjects.**

☐ Provisional Approval: the submitted *insert "project/study/proposal or other descriptive"* is approved pending development of the research plan (45CFR46.118), which must be reviewed before enrollment of subjects or collection of data can begin. Proposed date of review: __

☐ Conditional Approval: data collection of *insert "project/study/proposal or other descriptive"* can proceed after meeting the following conditions:

☐ Approval: approval of *insert "project/study/proposal or other descriptive"* is granted and data collection can proceed. In keeping with our Federalwide Assurance mandate, the IRB must conduct reviews at least annually for each project. This project will be reviewed again on *insert review date*.

☐ Approval Denied: approval of *insert "project/study/proposal or other descriptive"* is denied and data collection may not proceed for the following reasons:

3. Consent Procedures

The Institutional Review Board has determined that consent procedures:

☒ are not applicable to the project.

☐ must be reviewed on .

☐ are approved as submitted.

☐ are approved under the following conditions:

☐ are not approved for the following reasons:

4. Individually Identifiable Information Safeguards

The Institutional Review Board has determined that the safeguards planned for individually identifiable information:

☒ **are not applicable to the project**.

☐ must be reviewed on .

☐ are approved as submitted.

☐ are approved under the following conditions:

☐ are not approved for the following reasons:

5. Comments

On the basis of this review, the IRB has determined that the activities, as described in the submission, are exempt. The proposed activities involve the analysis of deidentified data for the purposes of improving the validity of tests, improving scoring, or improving measurement. These activities are not designed to develop or contribute to generalizable knowledge as described in 45 CFR 46.102(d). Additionally, the researchers cannot readily ascertain the identity of subjects as described in 45 CFR 46.102(f)(2). Therefore, these activities do not constitute human subjects research covered by 45 CFR 46.

6. IRB Signature(s):

06/27/2016
Date

Sandra L. Eyster
IRB Representative

Please keep in mind that any material changes made to the study or the study procedures require the submission of an updated IRB package.
